# Suicide Prevention in Youth

**DOI:** 10.1007/s11920-025-01620-w

**Published:** 2025-07-29

**Authors:** Cheryl A. King, Jane Harness, Alejandra Arango, Ewa Czyz

**Affiliations:** Department of Psychiatry, University of Michigan Medical School, Rachel Upjohn Building, 4250 Plymouth Road, Ann Arbor, MI 48109 USA

**Keywords:** Adolescent suicide risk, Suicide risk screening, Suicidal ideation, Suicide attempt warning signs, Therapeutic interventions, Social media

## Abstract

**Purpose of Review:**

This review presents new research pertinent to youth suicide prevention with a focus on suicide risk screening; therapeutic interventions, including Crisis Lifeline services; the identification of proximal risk or warning signs; and guidelines for youth discussions of suicide-related concerns on social media.

**Recent Findings:**

Universal screening for youth suicide risk is feasible in healthcare settings, identifies previously unrecognized risk, and has sufficient sensitivity and specificity for the prediction of suicide attempts. Recent large scale intervention trials have neither identified new effective treatments nor ways to make current treatments more effective. In other recent studies, however, youth and parents have independently identified suicidal communications, withdrawal from people and/or usual activities, and sleep problems as acute warning signs.

**Summary:**

Universal screening for youth suicide risk is recommended in healthcare settings. It identifies previously unrecognized risk at a reasonable cost. Regarding therapeutic interventions, additional research is needed to identify subgroups of youth that may benefit from specific interventions and to personalize these interventions for improved effectiveness in a way that is feasible and scalable in real world settings. Finally, research has identified acute or proximal warning signs for adolescent suicide attempts, and widescale dissemination of this information is recommended..

## Introduction

**Fig. 1:**
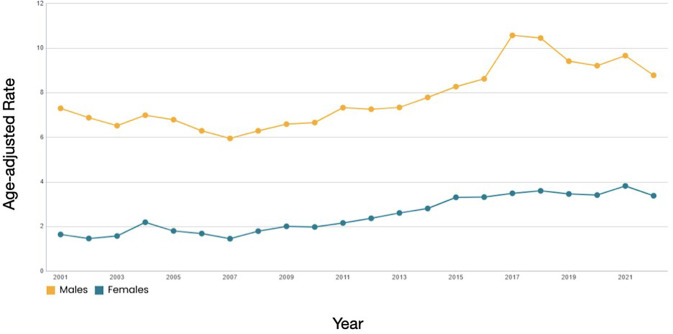
Trends in the age-adjusted suicide rate for youth, ages 10–19, by sex (2001 to 2022). Produced by the National Center for Injury Prevention and Control, Centers for Disease Control and Prevention (CDC) via the Web-based Injury Statistics Query and Reporting System (WISQARS), December 2024. Available from URL: www.wisqars.cdc.gov

Designated a national priority in the United States nearly a quarter century ago [1], suicide prevention continues to be a public health imperative. Suicide is currently the second leading cause of death among youth in the United States [2], and, as is evident in Fig. 1, the number of adolescents reporting suicidal thoughts and behaviors has risen significantly over the past 20 years [3]. This is particularly pronounced for identifiable subgroups such as Black youth, for whom the suicide rate has increased sharply (54% between 2018 and 2022) [4]. Despite substantial effort, we have been unable to move the youth suicide rate downward in a significant and sustained manner. Suicide is widely considered to be a preventable, albeit frustratingly intractable, public health problem.

Many scientists, including clinical investigators, are working to inform the development and implementation of evidence-based suicide prevention strategies. Facilitated by funding from federal agencies (e.g., National Institute of Mental Health, Centers for Disease Control and Prevention) and private foundations (e.g., American Foundation for Suicide Prevention), the breadth of initiatives under investigation are consistent with the wide angled public health approach described in *Suicide Prevention Resource for Action: A Compilation of the Best Available Evidence* [5]. These range from more universal prevention strategies, implemented in school, community, and healthcare settings, to more targeted clinical interventions for youth at high risk.

In this review, we focus on recently published research that meaningfully informs our collective efforts to prevent youth suicide. These studies focus on the screening and recognition of youth at risk for suicide in healthcare settings; therapeutic interventions provided in multiple settings as well as the more broadly available Crisis Lifeline services; and the identification of warning signs of acute proximal risk for suicide (applicable across school, community, and healthcare settings); We also present a new area of research investigation involving the development of guidelines for youth discussions of suicide-related issues on social media.

## Youth Suicide Risk Screening in Healthcare Settings

Youth suicide risk screening has the potential to save lives when implemented as one component of a clinical pathway that includes further assessment and referral for treatment. There is a growing evidence base regarding the strength of available suicide risk screening instruments (e.g. [6, 7]), and a consensus that universal screening for youth suicide risk can be effectively implemented in emergency departments and other healthcare settings [8].

The evidence in support of youth suicide risk screening in healthcare settings has grown substantially since early studies demonstrating the feasibility of universal screening and the ability of screening to detect previously unrecognized risk [9, 10]. Until recently, however, most studies of screening instruments were either cross-sectional, reporting screen sensitivity and sensitivity in terms of concurrent suicidal ideation (e.g. [9]), or they reported outcome data only on those who screened positive, limiting the possibility of examining predictive validity (e.g. [11]). With the relatively recent and large-scale NIMH-funded Emergency Department Screening for Teens at Risk for Suicide (ED-STARS) study, we now have a wealth of data enabling us to explore, prospectively, the suicide attempt outcomes associated with positive and negative screens [6].

Emergency Department-Based Suicide Risk Screening. Two recent ED-STARS research reports are particularly notable due to this study’s prospective research design and large, geographically diverse sample. Brent et al. (2023) compared the predictive validity of the Ask Suicide Screening Questions (ASQ [9]), and the Computerized Adaptive Screen for Youth Suicide Risk, (CASSY [6]) in relation to suicide attempt outcomes within three months of adolescents’ ED visits [7]. The ASQ is a 4-item self-report survey with a positive screen defined as a positive response or nonresponse to any one or more items. The CASSY is a computerized adaptive screen that includes three of the ASQ items and additional items that vary depending on responses to prior items (mean of 11 items per youth). In the ED-STARS validation study, 2,740 youth completed screenings and follow-ups. The performance of the ASQ and CASSY (e.g., sensitivity, specificity) did not differ significantly for patients who did not present with behavioral health chief complaints [7]; however, the CASSY performed significantly better for adolescents with behavioral health chief complaints. A choice between these two screening instruments requires balancing several considerations. The ASQ has the advantage of being in the public sector and readily available, whereas the CASSY requires computerized tablets and a licensing agreement (cost). Given the growing number of adolescents presenting to medical emergency departments with behavioral health and psychiatric concerns, the greater strength of the CASSY with this group may be an important consideration. As noted in the Blueprint for Youth Suicide Prevention [8], other tools such as the triage version of the Columbia Suicide Severity Rating Scale (C-SSRS [12]), have previously documented validity and are also available.

 The cost of universal suicide risk screening is a critically important issue for healthcare systems. A recent cost analysis of adolescent suicide risk screening in the ED was completed with the large and geographically diverse ED-STARS sample [13]. When the CASSY was used as a universal screen for all adolescent ED patients able to complete a self-report survey, the cost of screening per adolescent, including the cost of mental health follow-up evaluations, was $5.77 per adolescent. This micro-analysis considered site-specific personnel and resource costs from the time of the adolescent’s admission through their discharge or hospitalization, making the findings reasonably generalizable to other self-report screening tools. [It did not include the cost of CASSY software or program integration with electronic health record.] When analyzed only for those adolescents who presented with non-psychiatric chief complaints (adolescents who would not routinely be screened and receive mental health evaluations, as needed), the cost was only $2.60 per adolescent. Given that suicide is the second leading cause of death among adolescents, suicide risk screening would seem to be a justifiable service, particularly given the relatively low cost demonstrated in this study.

## Therapeutic Interventions

Comprehensive meta-analytic [22, 23] and systematic review [24, 25, 26] studies published in the past decade highlight existing interventions for youth with suicidal ideation or behavior that vary in treatment modality, targets, as well as evidence base supporting their effectiveness. A persistent challenge concerning intervening with adolescents at risk for suicide is that few psychosocial interventions have demonstrated to be effective [22, 27, 28]. To date, the treatment with most empirical support is Dialectical Behavioral Therapy (DBT-A) [29, 30], although even this well-established intervention showed benefits that were not sustained over time [29]. Yet, the prevalence of adolescent suicidal ideation and behavior clearly make them a treatment priority. In this section, we summarize recent randomized controlled trials (RCTs) in which the primary focus was prevention of suicidal ideation or suicidal behavior.

First, an RCT involving adolescents, ages 12–18, hospitalized for suicidal ideation and/or suicide attempts assessed the efficacy of a brief therapist-delivered intervention (As Safe As Possible or ASAP), a safety plan mobile application (BRITE app), and their combination (ASAP + BRITE app) [31]. The therapist delivered ASAP was grounded in an MI framework, focused on safety planning and skill acquisition, while the BRITE app included a personalized safety plan along with emotion regulation and distress tolerance skills. Moreover, as part of the ASAP and/or BRITE app, adolescents and their caregivers received two brief follow-up calls. Adolescents (*n* = 240) were randomized to one of four groups: ASAP with BRITE app, BRITE app, ASAP, or treatment as usual (TAU). There were no differences between the two interventions, either alone or together, and TAU on suicide attempts, suicidal ideation, non-suicidal self-injury, or suicidal events. In further moderation analyses, adolescents who were hospitalized for suicide attempts and assigned to BRITE app had a lower rate of follow-up suicide attempts. Taken together, the results were nuanced, suggesting that BRITE may lower suicide attempt risk for a subset of youth with recent attempts.

A second RCT was carried out nationwide via online recruitment with 565 adolescents, ages 13–16, with non-suicidal self-injury (NSSI) within the prior month and recent negative views of the self. In this study, youth were randomized to the web-based Project SAVE (Stopping Adolescent Violence Everywhere), a 30-minute self-administered single-session intervention drawing on elements of CBT, or to a 30-minute active control program with supportive therapy [32]. Project SAVE was perceived as acceptable; however, there were no group differences for the pre-registered outcomes of 3-month NSSI frequency or suicidal ideation or for post-intervention self-reported likelihood of future NSSI. Although adolescents randomized to Project SAVE reported post-intervention improvements in self-hatred and desire to stop future NSSI, overall results suggested that improvements in longer-term suicidal and non-suicidal self-injury outcomes may require additional or more intensive approaches.

A comparative effectiveness trial of a brief suicide crisis intervention based on Interpersonal Psychotherapy for Adolescents (IPT-A-SCI) for children and adolescents (*N* = 309) with depression, anxiety, or suicidal ideation/behavior showed no difference between the brief IPT-A-SCI, treatment as usual or waitlist on outcomes of interest including suicidal ideation, depression symptoms, or anxiety [33]. Youth were recruited in an outpatient depression and suicide clinic, and this intervention included 5 weekly 50-minute sessions and 4 follow up personal emails. Sessions focus on the development of a safety plan, evaluating interpersonal relationships, development and practice of coping strategies, and relapse prevention [33].

Finally, we describe two pilot studies exploring adjunctive interventions integrating follow-up contacts during high-risk care transitions. In the first pilot using a sequential multiple assignment randomized trial (SMART), 80 adolescent inpatients [34] were first randomized to a Motivational Interview-Enhanced Safety Plan (MI-SP), either alone or with daily post-discharge supportive text messages. Adolescents were re-randomized two weeks after discharge to an added booster call or to no call. Adolescents who received supportive text messages showed improved safety plan use, self-efficacy to refrain from suicidal action, and coping by support-seeking, as did youth randomized to booster calls. Supportive texts were also associated with lower intensity of daily suicidal urges and greater coping self-efficacy. Though needing additional empirical support, text messaging could offer a promising follow-up strategy and extend interventions, such as safety planning.

The second pilot RCT examined a text-based intervention for parents of adolescents seeking ED services for suicidal ideation or attempts ([35], under review). The automated text messages included two types: messages encouraging parents to follow through with recommendations for their adolescent (e.g., restricting lethal means) and messages supporting parents’ own well-being (e.g., coping tips). Parents (*n* = 120) were randomized to a control group or an intervention group consisting of one or both types of messages. These preliminary findings supported the feasibility and acceptability of this intervention and pointed to its positive impact on parental engagement in suicide prevention activities and lowering post-ED suicide attempts among adolescents. The unique focus on caregivers highlights the potential of low-burden parent-facing interventions as an additional strategy for lowering youth suicidal behavior during high-risk periods.

Altogether, there have been notable recent efforts toward building a stronger evidence-base for suicide-specific interventions for youth. Nevertheless, while demonstrating feasibility and acceptability, none of the recent large-scale RCTs reviewed showed an impact on suicidal ideation or attempts, and the two pilot trials require replication.

As the field looks toward addressing gaps in treatment approaches for adolescents at risk for suicide, we pose four recommendations. First, while recognizing the limitations of existing interventions, it may be that some treatment approaches, while not effective across the board, may in fact be beneficial for some youth. Though relatively infrequently tested in the suicide prevention literature [36], examining treatment moderators as part of RCTs could avoid prematurely discounting interventions that do not show overall effects and may inform how to personalize treatments [36]. For example, while the mobile BRITE app did not lower the suicide attempt risk overall, it was beneficial for those with recent attempts [31]. Second, as suicidal youth are not homogeneous with respect to risk profiles [37] or time-varying changes in risk states [38], a related consideration is to test adaptive interventions that specify how, when, and for whom interventions should be delivered. While this section described pilot work seeking to inform an adaptive intervention for youth at risk for suicide [34], large-scale studies will be necessary to guide progress in this area. Third, studies summarized in this section suggest a shift toward more scalable interventions that rely on technology to extend their reach [31, 34] or increase access to stand-alone support [32]. Nevertheless, caution is needed to scrutinize potential limitations of technology-based interventions, as such approaches may not necessarily lead to lasting improvement [32] or benefit all teens [31]. Finally, as we work to improve the effectiveness of preventive interventions, it is critically important to simultaneously address implementation issues, such that interventions are designed with features that improve intervention access and eventual uptake in real-world settings.

*Crisis Lifeline*. The 988 Crisis Lifeline is a nationally accessible service that offers live support via call, chat or text options. This service is considered to be a critical component of current suicide prevention efforts [39]. A recent study of Lifeline chatters’ views of the Lifeline’s perceived effectiveness revealed that 39% of chatters were minors [40] and 71.4% were 24 years old or younger. Two-thirds of chatters found the service helpful and nearly half reported a decrease in suicide-related concerns related to their chatting experience. Compared to adults, minors had a significantly higher odds of finding the chat helpful [40]. This converges with evidence from a 2022 study that analyzed data from crisis texters and found that the majority (i.e. 76%) were under 25 years old [41]. Highlighting the potential value of crisis support, 31% of texters 13 years or younger reported that they had no additional sources of help for their current crisis. Additionally, Hispanic, Black, and Native Hawaiian or Other Pacific Islander texters had lower rates of getting help from a therapist or doctor. Those who self-identified as belonging to a sexual or gender minority group and those reporting more than one race represented a greater proportion of texters compared to the general population. These findings highlight the potential benefit of the crisis texting resource for youth in general as well as for systematically marginalized groups, indicating its important role in advancing health equity. Additional research is needed that examines the effectiveness in relation to youth mental health and behavioral outcomes.

## Identification of Proximal Risk or Warning Signs

Researchers have begun to identify the signs of proximal risk for suicidal ideation and behavior in adolescents – those which are present during the 24-hours or less prior to the suicidal experience. Most prior research on youth suicide risk factors focused on distal or moderate- to short-term risk factors that occur in the months and years prior to a suicide attempt [14]. Although these studies provided information about *who* is at greatest risk, they did not help us understand *when* a youth may be at greatest risk [15, 16], which is critically important to caregivers, school personnel, and healthcare providers who face the stressful challenge of recognizing acute risk in time for potentially life-saving interventions.

A number of recent studies of proximal risk in adolescents have focused on risk for suicidal ideation and used technological advances such as ecological momentary assessments (EMA) and daily monitoring (e.g. [17]). Czyz et al.’s particularly noteworthy study focused on the prediction of next day suicidal ideation among adolescents following psychiatric hospitalization for suicide risk [18], which is a high-risk time with observed variability in day-to-day suicidal thinking [17, 19]. In this study, adolescents, who had been hospitalized for acute suicide risk, completed daily, text-based, surveys for four weeks following hospitalization. Using machine learning methods, the best-performing model for the prediction of next-day suicidal ideation was comprised of suicidal ideation, hopelessness, burdensomeness and suicide coping self-efficacy. Both risk factors over time (e.g., cumulative risk factor means over days) and proximal risk factors (e.g., past day experiences as compared to mean experiences) were important. Moreover, several combinations of risk factors were related to next-day suicide ideation, with suicide ideation duration as the most potent factor. Czyz et al. identified a reasonably good predictive model that did not include suicidal ideation, which may be important clinically given that some youth do not disclose suicidal thoughts. This study highlights the promise of machine learning to identify short-term risk for suicidal ideation. It should be noted, however, that the sample was small (*n* = 78) and primarily comprised of White adolescents, such that replication is warranted.

Moving beyond proximal risk for suicidal thoughts to proximal risk for suicide attempts, recent research has identified the 24-hour warning signs for adolescent suicide attempts [20]. In this study, 1,094 adolescents, recruited from geographically diverse pediatric emergency departments in the U.S., completed bi-weekly text message surveys for 18 months. These adolescents presented to the emergency departments with a wide range of psychiatric and nonpsychiatric presenting problems and were invited to participate in this prospective, community-based study if they had one or more suicide risk factors (e.g., depression). Adolescents who reported a suicide attempt during this follow-up period were interviewed regarding their thoughts, feelings/emotions, and behaviors/events during the 24-hours prior to their attempt (case period) and a comparison prior 24-hour period (control period). Their parents also participated in interviews reporting on adolescents’ behaviors and experiences during these periods. Interview data were available for 105 adolescents who reported suicide attempts. Using a within subject (case-crossover) design, suicidal communications, withdrawal from activities, and sleep disturbance emerged as key behavioral warning signs (Table 1). Additional affective, cognitive, and behavioral warning signs emerged from adolescent interviews and included, among others, self-hate, rush of feelings, emotional pain, less rage toward others, suicidal rumination, anger and hostility, perceived burdensomeness, and serious conflict with parents.

**Table 1:** Adolescent behavior and event warnings signs for suicide attempts

	Identified from Parent Interviews	Identified from Adolescent Interviews
**Adolescent Behavior or Event**		
Suicidal Communication	X	X
Withdrawal from People and/or Activities	X	X
Problem with Sleep	X	X
Serious conflict with parent		X
Negative romantic event		X
Other negative interpersonal event		X
Other negative life event (e.g., school-related)		X
Victimization (crime, dating violence, bullied)		X

NOTE: Data is for 105 adolescents who reported suicide attempts

These recent studies of proximal risk for adolescent suicidal ideation and suicide attempts provide important information for caregivers, school personnel and healthcare providers. Although replication of these studies is warranted, particularly with more diverse samples of youth, we now have evidence-based information to disseminate regarding proximal risk for suicidal ideation and suicide attempts among adolescents. A next challenge for our field will be learning how we can effectively help caregivers and clinicians identify these warning signs and respond in ways that are associated with positive outcomes for youth. It may be that the 24-hour behavioral warning signs for suicide attempts, including suicidal communications and withdrawal from others and activities, are most useful in this regard due to the difficulty of observing the adolescents’ thoughts and feelings. Regarding warning signs for suicidal ideation, whereas the combination of EMA or daily monitoring and methods for extraction of clinical data from electronic medical records has led to promising machine learning prediction models, these are not yet usable for the practical identification of acute risk in clinical settings. As a next step, we need to figure out how to extend these methods beyond the research context and make them usable for those who interact with youth. Finally, more frequent assessment of risk factors and machine learning techniques may open the door to adaptive interventions [21], which acknowledge that risk is multifactorial, and that youth may benefit from different interventions at different times.

## Social Media Guidelines for Youth Discussions of Suicide-Related Concerns

The potential benefits of social media, including connection and identity exploration, align with the developmental tasks of adolescence [42]. Nevertheless, social media may also heighten feelings of being left out and be a vehicle for cyberbullying and interpersonal conflict [43]. Youth may also be exposed to harmful pro-suicide and pro-self-harm content on social media [44], such as content encouraging suicide and self-harm or content that does not adhere to safe communication guidelines (such as descriptions of methods), which could contribute to suicide contagion/social transmission effects for some youth [45, 46].

The relationship between social media use and suicide was recently studied in a psychological autopsy study of 35 adolescents who died by suicide. According to the study informants (primarily parents and other next-of-kin), youth benefitted from support and recovery stories, but may have been harmed by dependence, harmful comparisons, triggers/imitation, cyberbullying, and cultivation of a “suicidal identity” [47]. The only time informants drew a direct relation between social media and the teen’s suicide was for youth who had been cyberbullied, with cyberbullying including the encouragement of suicidal behaviors, unsolicited proposals, or sexual coercion. Although there is no way to know whether these factors had any role in these adolescents’ suicides, these informants’ perspectives may provide important clues.

Social media use is associated with positive and negative experiences for youth with recent suicidal ideation and attempts, who have reported the benefit of accessing mental health and coping resources as well as the harm of excessive friendship expectations [48]. In a recent effort to maximize the benefits while minimizing the harms, an Australian-based program called #chatsafe was created to serve as a guide for safe peer-to-peer communication about suicide on social media. Developed using feedback from youth and suicide prevention experts [49], the guidelines address a variety of issues, including, among others, what to consider before sharing your own thoughts/experience with suicidal behavior and responding to someone who may be experiencing suicidal thoughts/behaviors [49]. In a preliminary study testing the acceptability, feasibility and safety of a social media campaign using #chatsafe guidelines, there were no serious adverse effects. Moreover, youth and young adults reported improvements in their willingness to intervene against suicide, perceived self-efficacy, confidence, and safety when communicating on social media about suicide. The #chatsafe guidelines and social media campaign are currently being tested in an RCT [50] assessing impact on young people’s safety and confidence when talking about suicide online.

Recommendations for the social media industry to promote help and prevent suicide include the importance of restricting self-harm and suicide related content by clear policies [51]. In a survey completed by 23 suicide prevention professionals and 43 young people, participants provided feedback on a range of social media considerations. There was moderate agreement about the industry using artificial intelligence (AI) to identify users at risk and promote helpful resources and strong support for legal requirements to provide education about communicating safely online in schools [51]. Many social media platforms already have community guidelines [52, 53, 54], which prohibit content promoting suicide, self-injury or eating disorders and users are directed to contact a helpline or reach out to a friend if they search for “suicide” related terms on some platforms.

## Conclusions

We have a growing evidence base to inform youth suicide prevention strategies; however, further research is needed to strengthen the effects of our emerging preventive strategies, tailor and improve them for identifiable subgroups of youth at risk, and to feasibly implement them in real world healthcare and community settings. Fortunately, the funding of suicide prevention research has grown substantially in recent years [55], including for high priority needs such as understanding and preventing Black youth suicide among Black youth [56, 57, 58]. Other subgroups of minoritized youth, such youth who identify as sexual and gender minorities (SGM; [59]) face unique stressors and are also at elevated risk, warranting further focused research [60]. We still need to understand which preventive strategies -- from screening to treatment to crisis response services -- work best for which subgroups of youth, as well as which can be disseminated widely and implemented feasibly in different settings, enabling us to address substantial mental health disparities.

Further research to inform youth suicide prevention is an urgent priority. Research alone, however, will not enable us to reach our suicide prevention goals. We also need to focus on the translation of research to policy and practice. This is likely to require developing and nurturing active working partnerships among researchers, healthcare professionals, healthcare and public policy changemakers, insurers, and leaders in pertinent youth-serving settings and institutions.

## Data Availability

No datasets were generated or analysed during the current study.

## Key References

- Brent, D.A., et al., *Prediction of suicide attempts and suicide-related events among adolescents seen in emergency departments.* JAMA Network Open, 2023. **6**(2): p. e2255986-e2255986. **In a prospective study of 2740 adolescents enrolled in pediatric emergency departments (EDs)**,** the Ask Suicide-Screening Questions (ASQ) and Computerized Adaptive Screen for Suicidal Youth (CASSY) performed similarly in predicting suicide attempts for patients with physical health chief complaints. The CASSY had greater predictive validity for patients with behavioral health complaints.**
- Czyz, E., et al., *Predicting short-term suicidal thoughts in adolescents using machine learning: developing decision tools to identify daily level of risk after hospitalization.* Psychological Medicine, 2023. **53**(7): p. 2982–2991. **This study focused on the prediction of adolescents’ next day suicidal ideation following psychiatric hospitalization for suicide risk. Using daily text-based surveys and machine learning methods**,** the best-performing model included suicidal ideation**,** hopelessness**,** burdensomeness and suicide coping self-efficacy.**
- Goldstein, T.R., et al., *Bridging gaps in care following hospitalization for suicidal adolescents: As Safe As Possible (ASAP) and BRITE App.* Journal of the American Academy of Child & Adolescent Psychiatry, 2024. Jul 18:S0890-8567(24)00361-7.doi: 10.1016/j.jaac.2024.06.008. Online ahead of print.**This randomized trial assessed the efficacy of a therapy (As Safe as Possible)**,** a safety plan mobile application (BRITE)**,** their combination**,** and treatment as usual in adolescents hospitalized for suicide risk. There were no differences on primary outcomes between the two interventions and TAU on suicide-related outcomes.**
- Grazier, K.L., et al., *The cost of universal suicide risk screening for adolescents in emergency departments.* International Journal of Environmental Research and Public Health, 2023. 20(19): p. 6843. **Using site-specific data from 10 pediatric emergency departments (EDs)**,** this study documented the low costs of universal suicide risk screening with adolescents relative to the cost of ED visits.**
- King, C.A., et al., *24-Hour warning signs for adolescent suicide attempts.* Psychological Medicine, 2024. **54**(7): p. 1272–1283. **This is a first empirical study of 24-hour warning signs for suicide attempts among adolescents. Using a case-crossover**,** within-subject design and adolesceent as well as parent interviews**,** multiple warning signs were identified that can be disseminated to families**,** clinicians**,** and the larger public.**
- Robinson, J., et al., *Testing the Impact of the# chatsafe Intervention on Young People’s Ability to Communicate Safely About Suicide on Social Media: Protocol for a Randomized Controlled Trial.* JMIR Research Protocols, 2023. **12**(1): p. e44300. **The #chatsafe program provides social media guidelines for safe suicide-related communications among peers. Representing innovative ongoing work**,** this article presents the protocol for a large-scale randomized controlled trial assessing the safety and positive impact of the #chatsafe guidelines with a social media campaign.**
